# Correction: Impact of the d^0^ transition metal on local structural transformations in disordered rock salt cathodes

**DOI:** 10.1039/d6sc90160c

**Published:** 2026-08-14

**Authors:** Tianyu Li, Otavio Marques, Tullio S. Geraci, Erick A. Lawrence, Arava Zohar, Jue Liu, Evans Avoka, Alexandra Navrotsky, Johanna Nelson Weker, Raphaële J. Clément

**Affiliations:** a Materials Department, University of California Santa Barbara Santa Barbara CA 93106 USA rclement@ucsb.edu; b Materials Research Laboratory, University of California Santa Barbara Santa Barbara CA 93106 USA; c Stanford Synchrotron Radiation Lightsource, SLAC National Accelerator Laboratory Menlo Park California 94025 USA; d Navrotsky Eyring Center for Materials of the Universe, Arizona State University Tempe AZ 85287 USA; e School of Molecular Sciences, Arizona State University Tempe AZ 85287 USA; f Neutron Scattering Division, Oak Ridge National Laboratory Oak Ridge Tennessee 37831 USA; g Ira A. Fulton School for Engineering of Matter, Transport and Energy, Arizona State University Tempe AZ 85287 USA

## Abstract

Correction for ‘Impact of the d^0^ transition metal on local structural transformations in disordered rock salt cathodes’ by Tianyu Li *et al.*, *Chem. Sci.*, 2026, **17**, 634–651, https://doi.org/10.1039/d5sc06959a.

The authors would like to detail the corrections listed below to the original article.

The primary correction to this article concerns the labeling of the octahedral and tetrahedral sites in the spinel-like “δ” phase. In particular, ordered spinel structures with stoichiometry Li_0.5_TMO_2_ (0 < *x* ≤ 1, TM: transition metal), equivalently written as LiTM_2_O_4_, comprise Li ions occupying tetrahedral sites that are corner-sharing with TM ions occupying octahedral sites. This cation arrangement can be described equivalently by placing the TM ions on the 16d Wyckoff sites and Li on the 8a sites, or the TM ions on the 16c sites and Li on the 8b sites. These two descriptions are symmetry equivalent, although the 16d/8a setting is more commonly used in the literature.

In the original version of this work, the Wyckoff sites were incorrectly labeled. Upon reexamining our Rietveld refinements of the synchrotron X-ray diffraction (SXRD) and neutron diffraction data, we confirmed that the refinements are consistent with preferential occupation of the 16d/8a (or, equivalently, the 16c/8b sites), with some amount of cation disorder. The Wyckoff site labels have therefore been corrected throughout the manuscript and SI.

In addition, we revised the description of the 16c octahedral Li environment in the spinel structure and updated the references used to infer the corresponding ^7^Li NMR shift in chemically delithiated and heat-treated Li_0.5_Mn_0.7_Zr_0.2_O_2_.

On page 642, the description of the 16c Li environment in the spinel phase should read as below, with the amended references:

‘Based on prior NMR studies of Li_2_MnO_3_ ^1,2^ and Li_2_Mn_2_O_4_,^3,4^ we estimate the shift of a Li in a 16c octahedral site in the spinel structure and surrounded by 6 nearest-neighbor Mn^4+^ (in adjacent 16d sites) connected *via* a double 90° Li–O–Mn pathway and 6 next-nearest-neighbor Mn^4+^ connected *via* a 180° pathway to be around 1100 ppm, while that for 16c Li surrounded by 12 Mn^3+^ (6 nearest-neighbors and 6 next-nearest neighbors) to be closer to 100 ppm. Hence, the observed intermediate 700 ppm shift is in line with that expected for 16c Li surrounded by 12 Mn with an average oxidation state of +3.86 as in Li_0.5_Mn_0.7_Zr_0.2_O_2_.’

On page 643, the sentence detailing the Li octahedral Wyckoff site label should read as below:

‘Furthermore, the low frequency tail observed below 400 ppm in all spectra collected after thermal activation could result from Li in octahedral (16c) or tetrahedral (8a) sites with one or more nearest-neighbor diamagnetic species (Zr^4+^, Li^+^ or vacancies).’

On page 645, the sentence detailing the Li and TM octahedral Wyckoff site labels should read as below:

‘This enhancement arises from the formation of spinel domains featuring 3D networks of 0-TM channels, where face-sharing octahedral (16c) and tetrahedral (8a) sites are alternately occupied by Li or are vacant, while TM ions reside on 16d octahedral sites, thus enabling efficient long-range Li-ion transport.’

In the revised SI for this work, in Table S3 the interchanged 16c and 16d Wyckoff site labels in the refinement of the synchrotron X-ray diffraction (SXRD) pattern for the heat-treated Li_0.5_Mn_0.7_Zr_0.2_O_2_ sample have been corrected. The caption for Table S3 has also been revised to clarify the spinel-domain structural model and to better distinguish this table from Table S5. Tables S4 and S5 were inadvertently swapped in the original version of the SI and have now been placed in the correct order, and in Table S5 the interchanged 16c and 16d Wyckoff site labels were corrected and the caption revised for clarity. In Table S6, the interchanged 16c and 16d Wyckoff site labels in the refinement of the neutron diffraction pattern for the heat-treated Li_0.5_Mn_0.7_Zr_0.2_O_2_ sample have been corrected.

The Royal Society of Chemistry apologises for these errors and any consequent inconvenience to authors and readers.

## Supplementary Material

SC-OLF-D6SC90160C-s001
