# Supplementary material for: Correction: Impact of the d0 transition metal on local structural transformations in disordered rock salt cathodes
Source: Chem Sci. 2026 Aug 14;17(33):16016–7. doi: 10.1039/d6sc90160c (PMC13475441; doi:10.1039/d6sc90160c)
Supplement: Supplementary file 1 [file SC-017-D6SC90160C-s001.pdf]

Supplementary Information (SI) for Chemical Science.
This journal is © The Royal Society of Chemistry 2026

Impact of the d^0 Transition Metal on Local Structural Transformations in Disordered Rocksalt Cathodes

Supplementary Information

Tianyu Li[a,b], Otavio Marques[c], Tullio S. Geraci[d,e], Erick A. Lawrence[a,b], Arava Zohar[a,b], Jue Liu[f], Evans Avoka[d,e], Alexandra Navrotsky[d,e,g], Johanna Nelson Weker[c] and Raphaële J. Clément[a,b]*

[a] Materials Department, University of California Santa Barbara, Santa Barbara, CA 93106, USA

[b] Materials Research Laboratory, University of California Santa Barbara, Santa Barbara, CA 93106, USA

[c] Navrotsky Eyring Center for Materials of the Universe, Arizona State University, Tempe, AZ 85287, USA

[d] School of Molecular Sciences, Arizona State University, Tempe, AZ 85287, USA

[e] Stanford Synchrotron Radiation Lightsource, SLAC National Accelerator Laboratory, Menlo Park, California 94025, USA

[f] Neutron Scattering Division, Oak Ridge National Laboratory, Oak Ridge, Tennessee 37831, USA

[g] Ira A. Fulton School for Engineering of Matter, Transport and Energy, Arizona State University, Tempe, AZ 85287, USA

* Corresponding Author: Raphaële J. Clément rclement@ucsb.edu

Methods

Materials synthesis. $Li_{1.1}Mn_{0.7}Zr_{0.2-x}Ti_xO_2$ were synthesized using a traditional solid-state synthesis method. Li_2CO_3 (Sigma, 99.99%), Mn_2O_3 (Alfa Aesar, 99%), TiO_2 (Alfa Aesar, 99%), ZrO_2 were ground together and pelletized. The pellets were sintered at 1100 °C for 12 h under argon (Ar) flow and then air-quenched to room temperature. Stoichiometric amounts of the precursors were used except for 3% mol of excess Li in the form of Li_2CO_3 to compensate for Li volatility during the calcination step.

Chemical delithiation and thermal activation of $Li_yMn_{0.7}Zr_{0.2-x}Ti_xO_2$ compounds. Chemically delithiated $Li_yMn_{0.7}Zr_{0.2-x}Ti_xO_2$ samples were prepared in an Ar-filled glovebox by reacting 200 mg of pristine DRX powder sample with nitronium tetrafluoroborate (NO_2BF_4) in a 1:1 molar ratio in a 20 mL acetonitrile solution. Reactions were conducted at room temperature for 3 days under constant stirring, and the reaction products were then filtered, thoroughly washed with acetonitrile, dried overnight in a vacuum oven at room temperature, and stored in an Ar-filled glovebox. For $Li_yMn_{0.7}Zr_{0.2-x}Ti_xO_2$ (x=0 and 0.05), due to the poor Li conductivity, the reaction temperatures were kept at 50 °C to ensure that the final product was homogeneous, with a final Li content of ~0.5. "Thermally activated" DRX samples were obtained by annealing the chemically delithiated samples at 400 °C under an Ar flow for 4h.

Structural and compositional characterization. The bulk chemical compositions (Li, Mn and Ti) of as-synthesized and chemically delithiated samples were determined via inductively coupled plasma (ICP) analysis using an Agilent 5800 ICP-OES instrument. The ICP results of the composition are listed in **Table S1**. As-prepared and chemically-delithiated samples were examined using synchrotron X-ray diffraction

(SXRD) at Beamline I11 at the Diamond Light Source (λ = 0.824104 Å). In-situ heating SXRD experiments were also conducted on selected samples. Chemically delithiated DRX samples were sealed in borosilicate capillaries under an Ar atmosphere. The capillaries were heated to 500 °C at a rate of 6 °C/min, with diffraction patterns collected every minute. Select samples were examined using time-of-flight (TOF) neutron diffraction and pair distribution function (PDF) analyses. The neutron diffraction patterns were collected at 300 K on the Nanoscale-ordered Materials Diffractometer (NOMAD) at the Spallation Neutron Source (SNS), Oak Ridge National Laboratory (ORNL). For in-situ heating measurement, chemically delithiated DRX samples were sealed in vanadium cans and heated from room temperature to 400 °C at a rate of 10 °C/min. During heating, the temperature was held at 25, 100, 200, 300, and 400 °C for 2 hours each to allow for data collection. Rietveld refinements on diffraction patterns and refinements on PDFs were performed using TOPAS 7[1].

Scanning electron microscopy (SEM) images were obtained using a Thermo Fisher Apreo C LoVac SEM instrument, an accelerating voltage of 5 keV, and a current of 0.4 nA.

X-ray absorption spectroscopy (XAS) measurements were conducted at Mn and Ti K-edges at SSRL Beamline 4-3, and at the Zr K-edge at SSRL Beamline 9-3. XAS data were collected in transmission and fluorescence modes, using a beam spot size of HxV ≈ 4 x 1 mm^2. Beam damage was not observed even after extended exposure of the samples to the beam. Reduction of the Mn, Ti, and Zr K-edge data was performed using the Athena software. Mn, Ti, and Zr K-edge extended X-ray absorption fine structure (EXAFS) data were collected using a wavenumber $k = 14$ $Å^{-1}$ in step scan mode. Fourier transformation of the EXAFS ($\chi(R)$) data was conducted over the range $2 < k < 12$ $Å^{-1}$

using a Hanning window function with dk = 2.0 $Å^{-1}$. Mn K-edge FT-EXAFS results were analyzed using the Artemis IFEFFIT-based code. Only single scattering paths in the 1.0 < R < 3.1 $Å^{-1}$ $\chi(R)$ range were considered, consisting of Mn – O, Mn – M (Li/Mn/Ti/Zr), and Mn – O' correlations, being the latter relative to next Mn – O coordination shell. The EXAFS model includes bond length (R), coordination number (N), and disorder (σ^2) parameters for Mn – O and Mn – M scattering paths. Due to the limited data range and number of independent variables available for the fit, only R and σ^2 were considered for Mn – O'.

Solid-state nuclear magnetic resonance (NMR) spectra were acquired at 2.35 T (100 MHz for ^1H) using a wide bore Bruker BioSpin spectrometer equipped with a DMX 500 MHz console and a custom-made 1.3 mm, single channel broadband magic angle spinning (MAS) probe tuned to ^7Li (38.9 MHz). ^7Li NMR spectra were obtained using a rotor-synchronized spin–echo sequence (90°-t_R–180°-t_R) with 90° radio frequency pulses of 0.45 μs. The samples were spun at 50 kHz MAS using dry nitrogen. ^7Li chemical shifts were externally referenced against a 1 M aqueous LiCl solution (δ_{iso} = 0 ppm). A recycle delay of 1 s was used throughout, sufficiently long for all paramagnetic ^7Li spins within the DRX structure to re-equilibrate between scans. The NMR data was processed using the Bruker TopSpin 3.6.0 software, and spectra were fitted using the DMfit software[2].

Calorimetry measurements

The enthalpy of formation from oxides and O_2 of chemically delithiated DRX compounds was measured before and after thermal activation by high temperature oxide melt drop solution calorimetry using a Calvet DC Alexsys calorimeter from Setaram Inc. Samples, ranging in size from 2 to 8 mg, were pressed into pellets using a die press. Each pellet

was then dropped from room temperature into molten sodium molybdate ($3Na_2O•4MoO_3$) solvent at 800 °C in the calorimeter. The sample was dissolved in and oxidized by the solvent. Oxidizing conditions were maintained with 35 mL/min of flushing and 4 mL/min of bubbling with $O_{2(g)}$. Bubbling of oxygen into the melt was also used to control oxygen fugacity, stir the solvent to aid sample dissolution and prevent local saturation. Calibrations were performed by measuring the heat effect of dropping high purity corundum (α-Al_2O_3) without solvent as previously described[3]. Calibrations were averaged over 12 samples measured over a 3-4 month period. The procedures described here are standard for Alexsys calorimeters[4–7]. Detailed thermochemical cycles and literature data used for calculation of the enthalpies of formation are presented below:

Reaction	ΔH_{rxn} (kJ/mol)
$Li_xM_yMn_zO_{2\,(s,298\,K)} \rightarrow \frac{x}{2}\,Li_2O_{(sol'n,1073\,K)} + y\,MO_{2\,(sol'n,1073\,K)} + \frac{z}{2}\,Mn_2O_{3\,(sol'n,1073\,K)} + \left(\frac{-x}{4} - y - \frac{3}{4}z + 1\right)O_{2\,(g,1073\,K)};\ M = Ti, Zr$	$\Delta H_1 = \Delta H_{ds,1073K}$
$Li_2O_{(s,298\,K)} \longrightarrow Li_2O_{(sol'n,1073\,K)}$	$\Delta H_2 = -77.21 \pm 2.44$
$TiO_{2\,(s,298\,K)} \longrightarrow TiO_{2\,(sol'n,1073\,K)}$	$\Delta H_3 = 73.37 \pm 0.36$
$ZrO_{2\,(s,298\,K)} \longrightarrow ZrO_{2\,(sol'n,1073\,K)}$	$\Delta H_4 = 29.2 \pm 1.6$
$Mn_2O_{3\,(s,298\,K)} \longrightarrow Mn_2O_{3\,(sol'n,1073\,K)}$	$\Delta H_5 = 175.79 \pm 1.38$
$O_{2\,(g,298\,K)} \longrightarrow O_{2\,(g,1073\,K)}$	$\Delta H_6 = 25.27 \pm 0.2527$
$\frac{x}{2}Li_2O_{(s,298K)} + y\,MO_{2(s,298K)} + \frac{z}{2}Mn_2O_{3(s,298K)} + \left(\frac{-x}{4} - y - \frac{3z}{4} + 1\right)O_{2(g,298K)} \rightarrow Li_xM_yMn_zO_{2(s,298K)};\ M = Ti, Zr$	$\Delta H_{f,ox\ 298\ K}$
$\Delta H_{f,ox} = \frac{x}{2}\Delta H_2 + y\,\Delta H_{3\ or\ 4} + \frac{z}{2}\,\Delta H_5 + \left(\frac{-x}{4} - y - \frac{3}{4}z + 1\right)\Delta H_6 - \Delta H_1$	

Electrochemical testing

The electrochemical performance of the materials of interest was tested in CR2023-type coin cells. Cathode films were prepared via dry rolling and cells were assembled in an Ar-filled glovebox. For the preparation of nanosized DRX electrodes, as-synthesized $Li_{1.1}Mn_{0.7}Zr_{0.2}O_2$ DRX powders were milled with carbon Super C65 in a 7:2 ratio at 400 rpm for 6 h using an Ar-filled jar and a planetary ball mill. The carbon-coated DRX

powders were then ground with polytetrafluoroethylene (PTFE) binder in a 9:1 weight ratio and the resulting mixtures were rolled out into films. For the preparation of micrometer-sized DRX electrodes (no particle downsizing), vacuum-dried DRX powders were ground with carbon C65, and PTFE binder in a 7:2:1 weight ratio, and the mixtures were rolled out into films. The films were then punched into 6.35 mm diameter discs weighing 2.5–3 mg each. Half-cells were then assembled using the cathode film, a Li metal counter electrode, a 1 M $LiPF_6$ in ethylene carbonate and dimethyl carbonate (EC/DMC with a 1:1 volume ratio) electrolyte, and a Celgard 2325 separator.

Table S1. Target compositions of $Li_yMn_{0.7}Zr_{0.2-x}Ti_xO_2$ DRX compounds of interest to this work, and their actual compositions from ICP before and after chemical delithiation.

Pristine DRX target formula	ICP formula	ICP formula after chemical delithiation
$Li_{1.1}Mn_{0.7}Zr_{0.2}O_2$	$Li_{1.133}Mn_{0.7}Zr_{0.196}O_2$	$Li_{0.535}Mn_{0.7}Zr_{0.204}O_2$
$Li_{1.1}Mn_{0.7}Zr_{0.15}Ti_{0.05}O_2$	$Li_{1.143}Mn_{0.7}Zr_{0.148}Ti_{0.044}O_2$	$Li_{0.524}Mn_{0.7}Zr_{0.151}Ti_{0.054}O_2$
$Li_{1.1}Mn_{0.7}Zr_{0.1}Ti_{0.1}O_2$	$Li_{1.121}Mn_{0.7}Zr_{0.11}Ti_{0.106}O_2$	$Li_{0.552}Mn_{0.7}Zr_{0.108}Ti_{0.109}O_2$
$Li_{1.1}Mn_{0.7}Zr_{0.05}Ti_{0.15}O_2$	$Li_{1.154}Mn_{0.7}Zr_{0.059}Ti_{0.143}O_2$	$Li_{0.513}Mn_{0.7}Zr_{0.054}Ti_{0.149}O_2$
$Li_{1.1}Mn_{0.7}Ti_{0.2}O_2$	$Li_{1.128}Mn_{0.7}Ti_{0.206}O_2$	$Li_{0.576}Mn_{0.7}Ti_{0.206}O_2$

Table S2. Structural parameters from Rietveld refinement of the neutron powder diffraction pattern collected on as-synthesized $Li_{1.1}Mn_{0.7}Zr_{0.2}O_2$.

Compound: $Li_{1.1}Mn_{0.7}Zr_{0.2}O_2$ R_{wp}=4.551%

Space Group: $Fm\bar{3}\,m$

***a*=*b*=*c*=**4.2203(1)

site	atom	x	y	z	occupancy	beq
4a	Li	0	0	0	0.55	1.31(2)
4a	Mn	0	0	0	0.35	1.31(2)
4a	Zr	0	0	0	0.1	1.31(2)
4b	O	0.5	0.5	0.5	1	1.63(2)

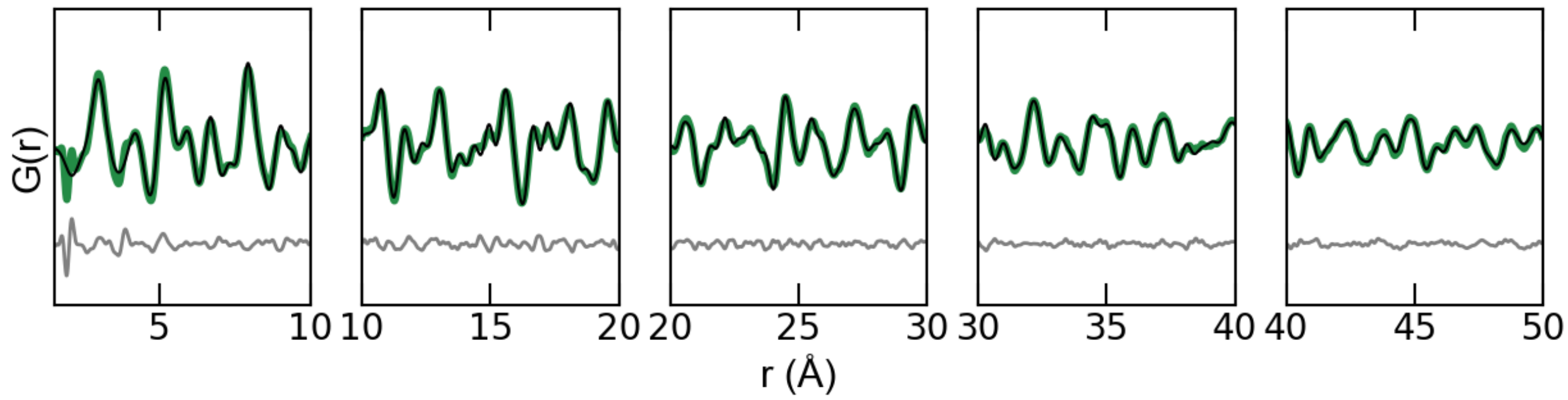

Figure S1. Small box fits of time-of-flight neutron PDF data collected on as-synthesized $Li_{1.1}Mn_{0.7}Zr_{0.2}O_2$ using a cation-disordered cubic rock salt model over different lengthscales.

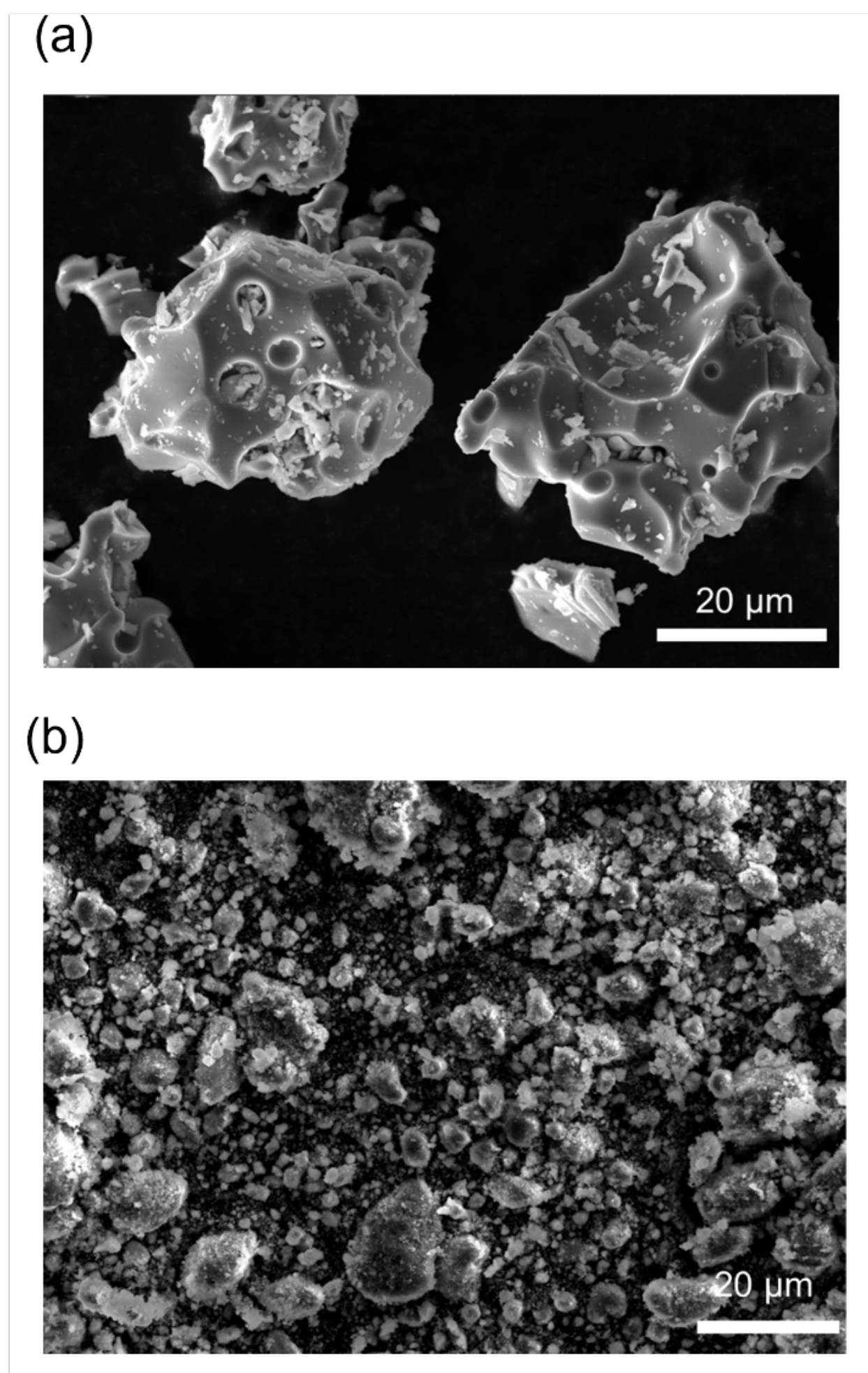

Figure S2. SEM images of (a) as-synthesized $Li_{1.1}Mn_{0.7}Zr_{0.2}O_2$ and (b) $Li_{1.1}Mn_{0.7}Zr_{0.2}O_2$ ball-milled with carbon Super C65 at 400 rpm for 6h.

Table S3. Structural parameters obtained from Rietveld refinement of the SXRD pattern collected on chemically-delithiated $Li_{0.5}Mn_{0.7}Zr_{0.2}O_2$ after a heat treatment at 400 °C for 4h. A "spinel domain" structural model was used for the refinement where the peak shapes of the hkl, l odd and l even reflections were separately refined. Li was excluded from the refinement due to its low X-ray scattering cross section. To reduce the complexity of the refinement, the Mn/Zr ratio for 16c and 16d octahedral site was constrained to 7/2, and the total occupancy of the octahedral sites was constrained to be 0.9 based on the actual chemical composition.

Compound: 400 ℃ thermally relaxed $Li_{0.5}Mn_{0.7}Zr_{0.2}O_2$ R_{wp}=3.67%

Space Group: $Fd\bar{3}m$

***a*=*b*=*c*=**8.19843(2)

site	atom	x	y	z	occupancy	beq
16d	7/9Mn, 2/9Zr	0.5	0.5	0.5	0.836(2)	2.24(2)
16c	7/9Mn, 2/9Zr	0	0	0	0.064(2)	2.24(2)
8a	Mn	0.125	0.125	0.125	0.0074(2)	0.9(6)
8b	Mn	0.375	0.375	0.375	0.0033(1)	0.9(6)
32e	O	0.2614(2)	0.2614(2)	0.2614(2)	1	2.75(5)

Table S4. Structural parameters from Rietveld refinement of the synchrotron XRD pattern collected on $Li_{0.5}Mn_{0.7}Zr_{0.2}O_2$ heat treated at 400 °C for 4h. DRX structure model is used for the refinement.

Compound: 400 ℃ thermally relaxed $Li_{0.5}Mn_{0.7}Zr_{0.2}O_2$ R_{wp}=12.001%

Space Group: $Fm\bar{3}m$

a=b=c=4.09920(3)

site	atom	x	y	z	occupancy	beq
4a	Li	0	0	0	0.55	2.17(5)
4a	Mn	0	0	0	0.359(4)	2.17(5)
4a	Zr	0	0	0	0.081(4)	2.17(5)
4b	O	0.5	0.5	0.5	1	3.17(5)

Table S5. Structural parameters obtained from Rietveld refinement of the SXRD pattern collected on chemically-delithiated $Li_{0.5}Mn_{0.7}Zr_{0.2}O_2$ after a heat treatment at 400 °C for 4h. A single phase spinel structural model was used for the refinement. Li was excluded from the refinement due to its low X-ray scattering cross section. To reduce the complexity of the refinement, the Mn/Zr ratio for 16c and 16d octahedral sites was constrained to 7/2 and the total occupancy of the octahedral site was constrained to 0.9 based on the actual chemical composition.

Compound: 400 ℃ thermally relaxed $Li_{0.5}Mn_{0.7}Zr_{0.2}O_2$ R_{wp}=11.41%

Space Group: $Fd\bar{3}m$

a=b=c=8.1984(6)

site	atom	x	y	z	occupancy	beq
16d	7/9Mn, 2/9Zr	0.5	0.5	0.5	0.566(2)	2.34(5)
16c	7/9Mn, 2/9Zr	0	0	0	0.334(2)	2.34(5)
8a	Mn	0.125	0.125	0.125	0.0081(2)	1(2)
8b	Mn	0.375	0.375	0.375	0.0023(5)	1(2)
32e	O	0.248(3)	0.248(3)	0.248(3)	1	2.92(8)

Table S6. Structural parameters from Rietveld refinement of the NPD pattern collected on $Li_{0.5}Mn_{0.7}Zr_{0.2}O_2$ heat treated at 400 °C for 4h. Spinel domain structure model is used for the refinement. Two sets of peak shapes for the hkl l odd and l even sets of reflections are defined. Best result is given when 16d site is set to be only occupied by Mn, and Zr, Mn, Li occupancies are refined at 16c site.

Compound: 400 ºC thermally relaxed $Li_{1.1}Mn_{0.7}Zr_{0.2}O_2$ R_{wp}=4.38%

Space Group: $Fd\bar{3}m$

***a*=*b*=*c*=**8.1894(8)

site	atom	x	y	z	occupancy	beq
16d	Mn	0.5	0.5	0.5	0.71(5)	0.21(3)
16c	Mn	0	0	0	0.003(2)	0.21(3)
16c	Zr	0	0	0	0.209(2)	0.21(3)
16c	Li	0	0	0	0.3842(2)	0.21(3)
8a	Li	0.125	0.125	0.125	0.257(8)	0.9(6)
32e	O	0.2624(2)	0.2624(2)	0.2624(2)	1	0.35(2)

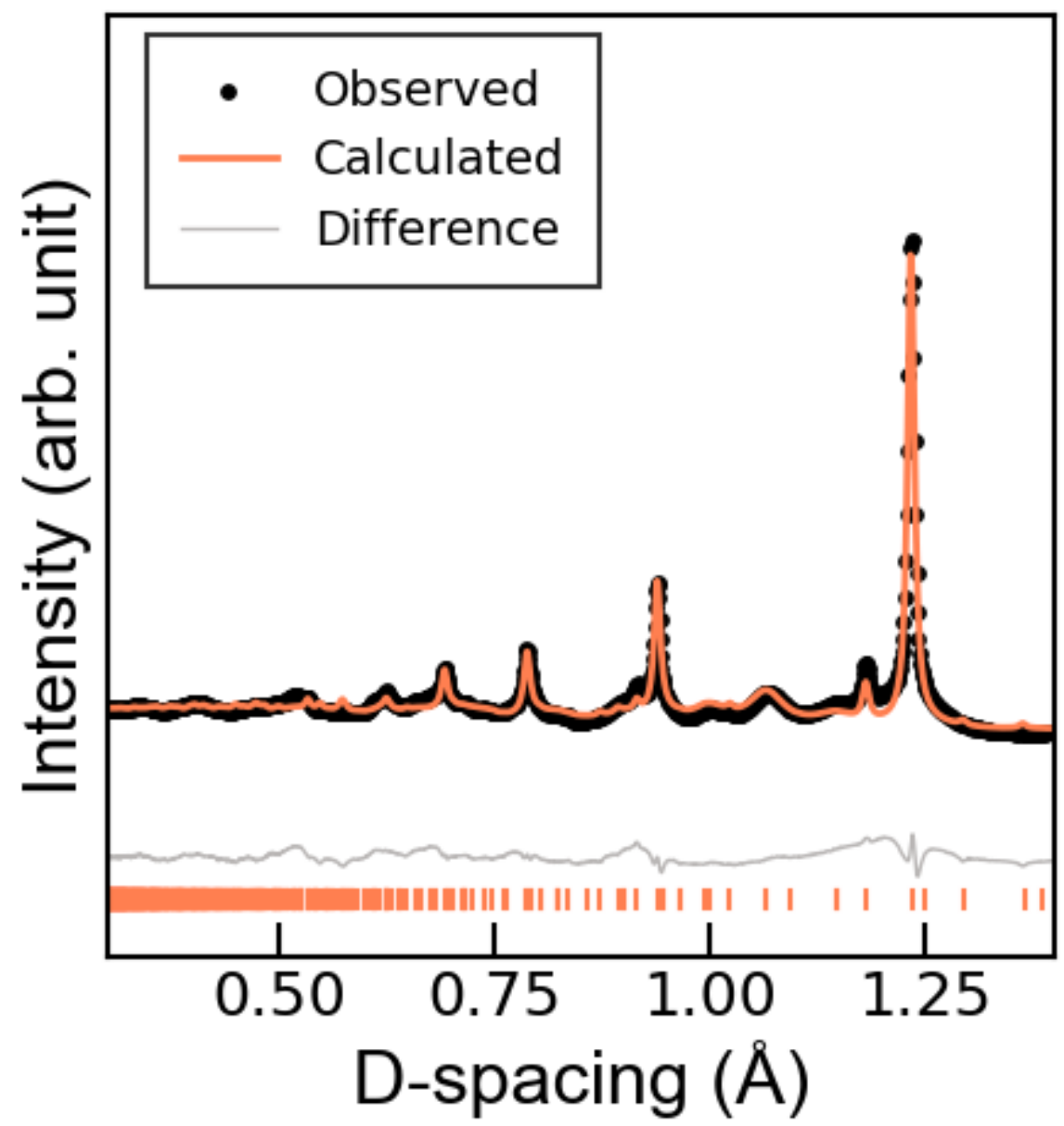

Figure S3. Neutron diffraction pattern, as well as Rietveld refinement fit of $Li_{0.5}Mn_{0.7}Zr_{0.2}O_2$ after thermal treatment. A single spinel phase model is used, with two sets of peak shapes for the hkl l odd and l even sets of reflections.

Note S1 — Fits of Mn K-edge EXAFS data obtained on $Li_{0.5}Mn_{0.7}Zr_{0.2}O_2$ and $Li_{0.5}Mn_{0.7}Ti_{0.2}O_2$ before and after thermal activation

More quantitative information about the evolution of the Mn local environments in both $Li_{0.5}Mn_{0.7}Zr_{0.2}O_2$ and $Li_{0.5}Mn_{0.7}Ti_{0.2}O_2$ upon a 400 °C heat treatment can be obtained from fits of the Mn K-edge EXAFS data. The fits were performed using a simplified cubic rock salt structural model where Mn fully occupies the octahedral cation sites (as in MnO). Here, the average number of Mn–M correlations obtained from the fits provides insight into the average Mn-M coordination environment. Fits were conducted over the 1.0 to 3.1 Å range, encompassing both nearest-neighbor (Mn–O) and next-nearest-neighbor (Mn–M) correlations. It is important to note that, given the simplified structural model used for the fits where Ti or Zr are replaced by Mn, next-nearest-neighbor Mn–M coordination numbers are systematically overestimated for $Li_{0.5}Mn_{0.7}Zr_{0.2}O_2$ and underestimated for $Li_{0.5}Mn_{0.7}Ti_{0.2}O_2$. Despite this, trends in the fitting results before and after the heat treatment are meaningful since the DRX composition does not change during heating to 400°C. Fits of the Mn K-edge EXAFS data collected on $Li_{0.5}Mn_{0.7}Zr_{0.2}O_2$ and $Li_{0.5}Mn_{0.7}Ti_{0.2}O_2$ powder samples before and after the heat treatment are shown in **Figure S4**, and the resulting parameters from the fit are listed in **Table S7**. Average Mn–O and Mn–M bond distances are around 1.9 Å and 2.9 Å, respectively, for all of the samples, which is consistent with the values obtained from Rietveld refinements of the diffraction data. The Mn–O coordination number is also close to six for all of the samples, confirming that Mn species remain octahedrally-coordinated throughout. $\sigma^2_{Mn\text{-}O}$ and $\sigma^2_{Mn\text{-}M}$ decrease after the heat treatment, suggesting that heating reduces the lattice distortions in both systems. As mentioned in the main text, the increase in the intensity of the Mn–M peak after heating, for both $Li_{0.5}Mn_{0.7}Zr_{0.2}O_2$ and $Li_{0.5}Mn_{0.7}Ti_{0.2}O_2$, is consistent with an increase

in the number of Mn–M (M = Mn, Zr, Ti) next-nearest-neighbor correlations, i.e., MnO_6–MO_6 clustering.

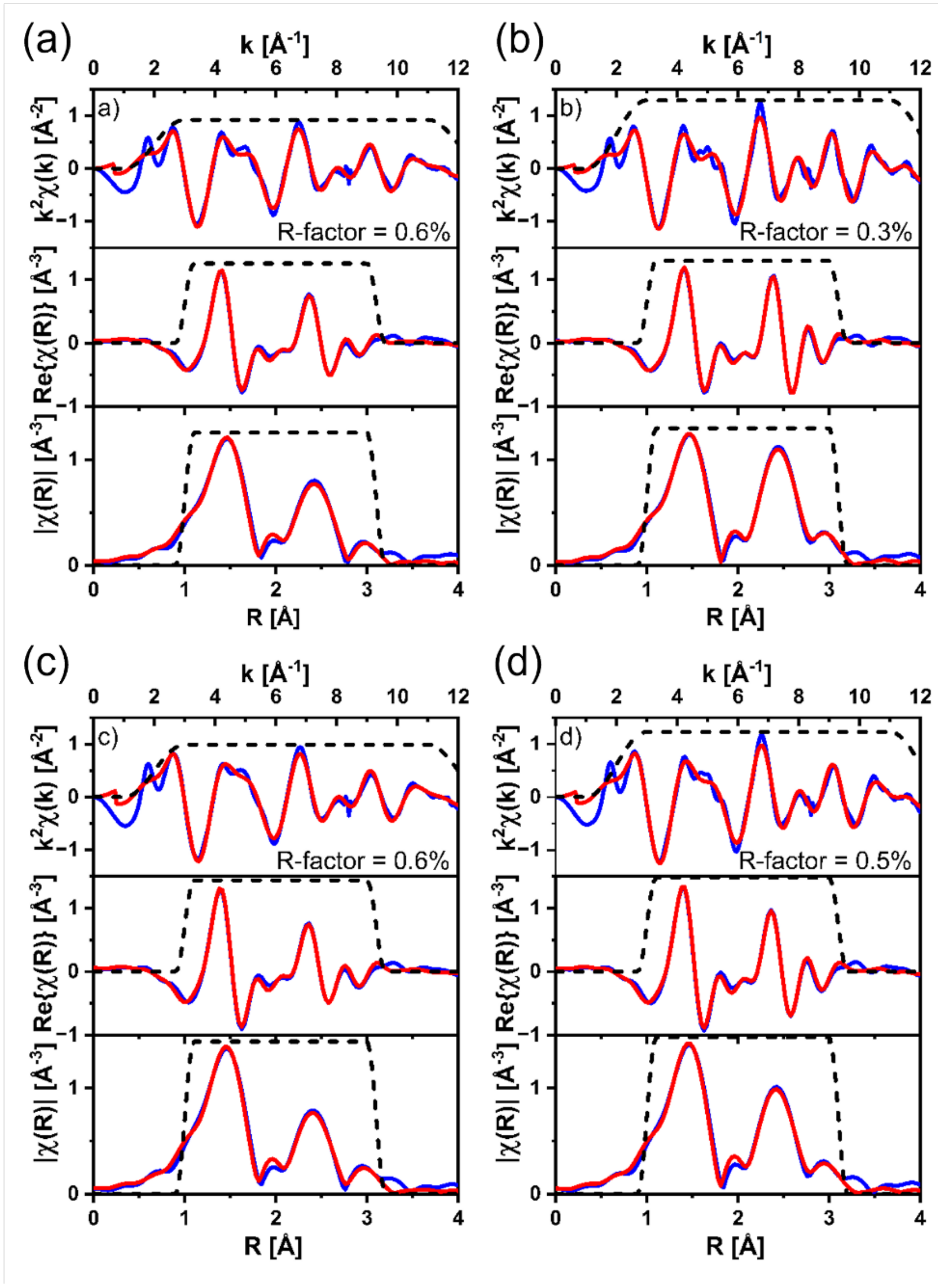

Figure S4. Fits of the Mn K-edge EXAFS data collected on chemically-delithiated $Li_{0.5}Mn_{0.7}Ti_{0.2}O_2$ (a) before and (b) after a 400 °C heat treatment for 4 h, and on chemically-delithiated $Li_{0.5}Mn_{0.7}Zr_{0.2}O_2$ (c) before and (d) after the heat treatment. The results are plotted in χ(k) (top panel), Re{χ(R)} (middle panel), and |χ(R)| (bottom panel), with the experimental data shown in blue and fit in red. The fitting ranges are indicated using Hanning window functions (black dashed line) with ∂k = 2.0 $Å^{-1}$ and ∂R = 0.2 Å. An amplitude reduction factor of 0.33 was extracted from a fit of EXAFS data obtained on a Mn_2O_3 standard (not shown).

Table S7. Structural parameters obtained from fits of the Mn K-edge EXAFS data collected on $Li_{0.5}Mn_{0.7}Zr_{0.2}O_2$ and $Li_{0.5}Mn_{0.7}Ti_{0.2}O_2$ samples before and after a 400 °C heat treatment for 4 h. R, N, and σ represent the interatomic distance, coordination number, and square variation in the EXAFS path length, respectively.

Sample Name	R_{Mn-O} (Å)	N_{Mn-O}	σ_{Mn-O} (Å^2)	R_{Mn-M} (Å)	N_{Mn-M}	σ_{Mn-M} (Å^2)
$Li_{0.5}Mn_{0.7}Zr_{0.2}O_2$	1.899(5)	6.4(5)	0.00268(8)	2.893(8)	5.1(1.3)	0.006(2)
$Li_{0.5}Mn_{0.7}Zr_{0.2}O_2$ after heat treatment	1.897(5)	6.3(4)	0.00202(8)	2.894(6)	6.5(1.3)	0.005(1)
$Li_{0.5}Mn_{0.7}Ti_{0.2}O_2$	1.899(5)	6.0(4)	0.00248(8)	2.904(7)	6.3(1.0)	0.007(1)
$Li_{0.5}Mn_{0.7}Ti_{0.2}O_2$ after heat treatment	1.904(4)	5.9(3)	0.00174(6)	2.908(4)	7.6(7)	0.0047(7)

Note S2 — Additional details on NPDF analysis of $Li_{0.5}Mn_{0.7}Zr_{0.2}O_2$ and $Li_{0.5}Mn_{0.7}Ti_{0.2}O_2$ before and after thermal activation

NPDF patterns collected on chemically-delithiated $Li_{0.5}Mn_{0.7}Zr_{0.2}O_2$ and $Li_{0.5}Mn_{0.7}Ti_{0.2}O_2$ before and after a 400 °C heat treatment for 4h are shown in **Figures S5** and **S6**, respectively. The positive peak intensities are dominantly attributed to Mn–Mn and O–O correlations, whereas negative intensities are primarily assigned to Mn–O correlations within the structure. The first negative peak at 1.9 Å, corresponding to Mn–O nearest-neighbor correlations, remains unchanged for both systems upon the heat treatment, indicating that the MnO_6 octahedra are mostly unaffected by the transformation, consistent with the Mn K-edge EXAFS results.

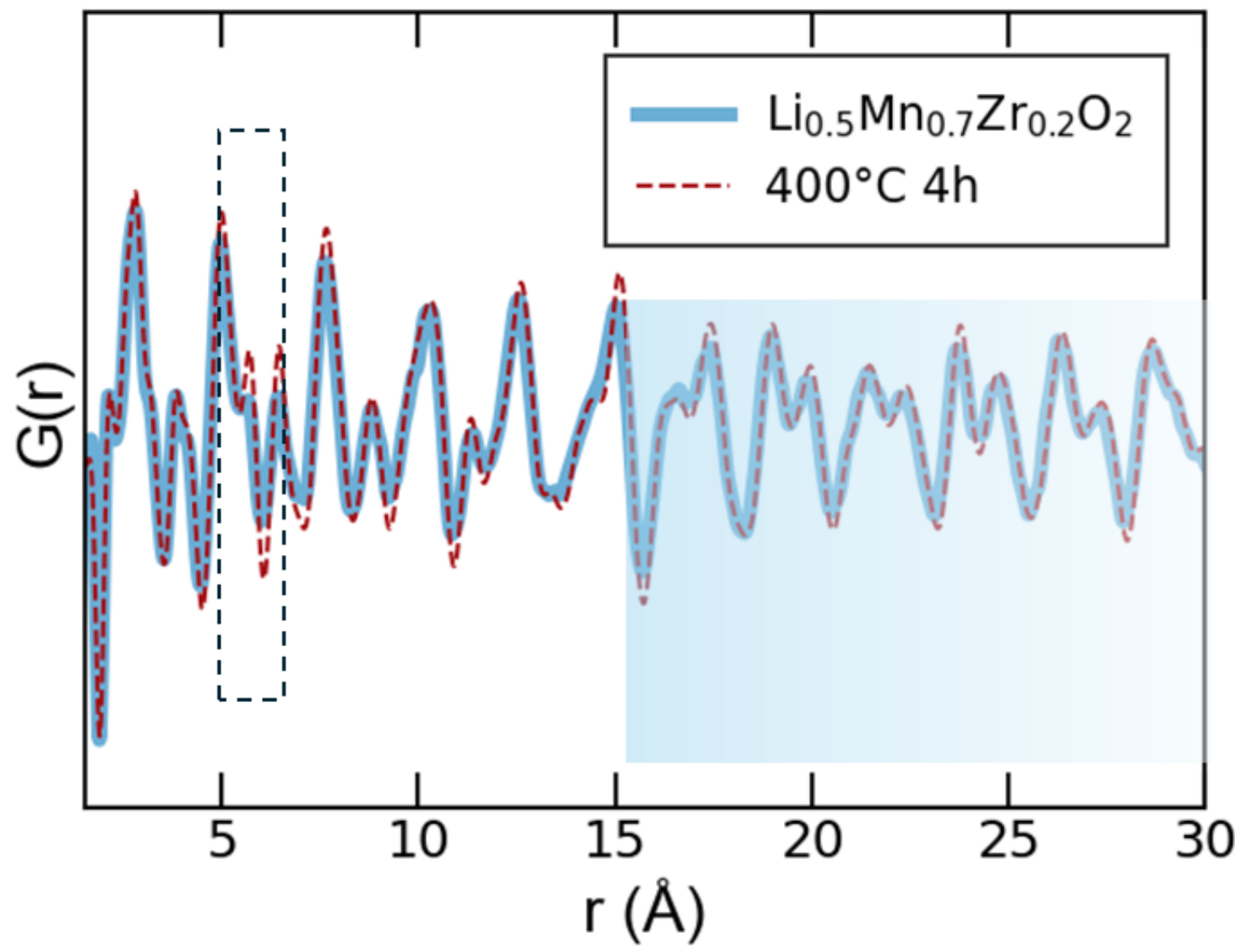

Figure S5. Neutron PDF of chemically delithiated DRX $Li_{0.5}Mn_{0.7}Zr_{0.2}O_2$ before and after the heat treatment.

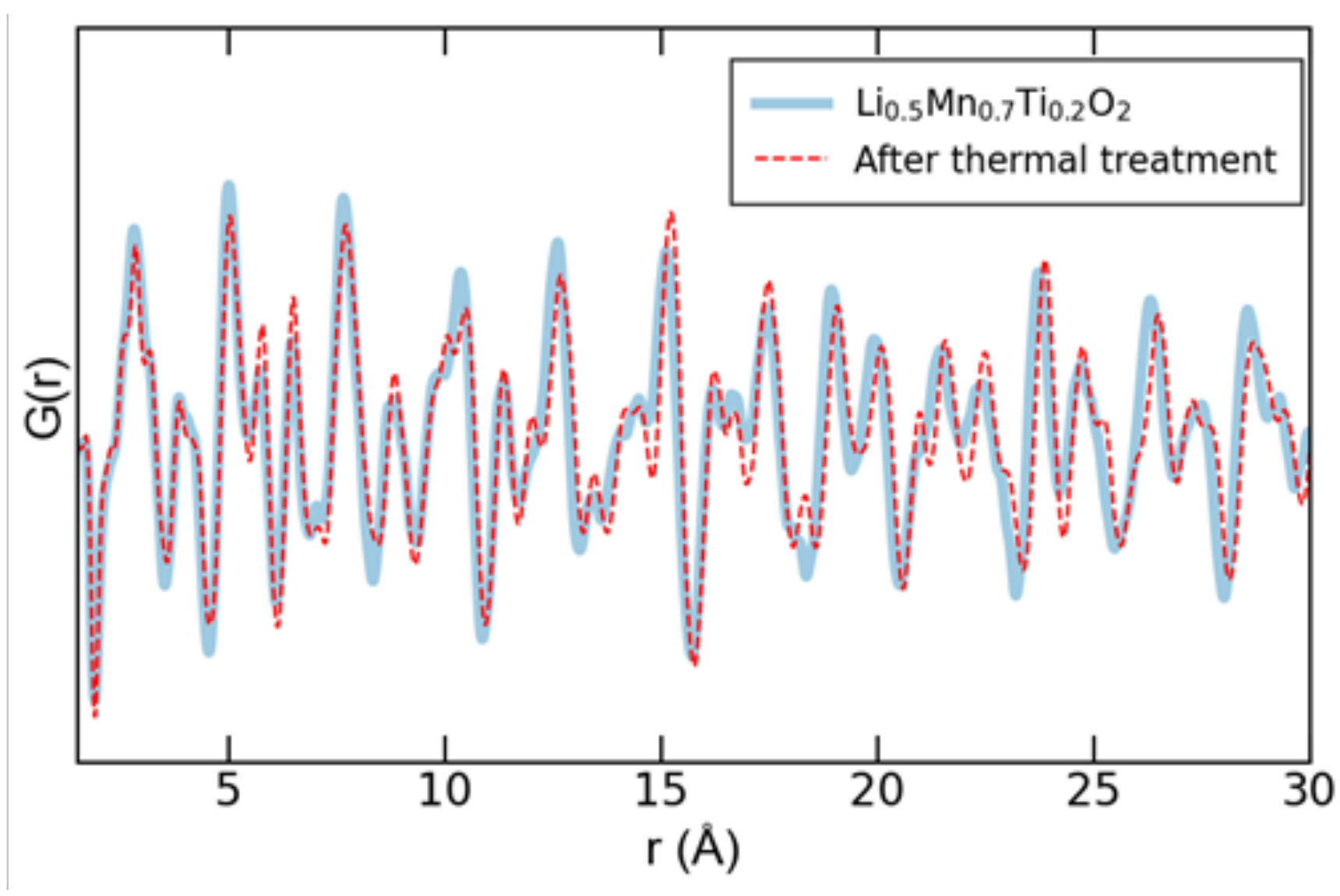

Figure S6. Neutron PDF of chemically delithiated DRX $Li_{0.5}Mn_{0.7}Ti_{0.2}O_2$ before and after thermal treatment.

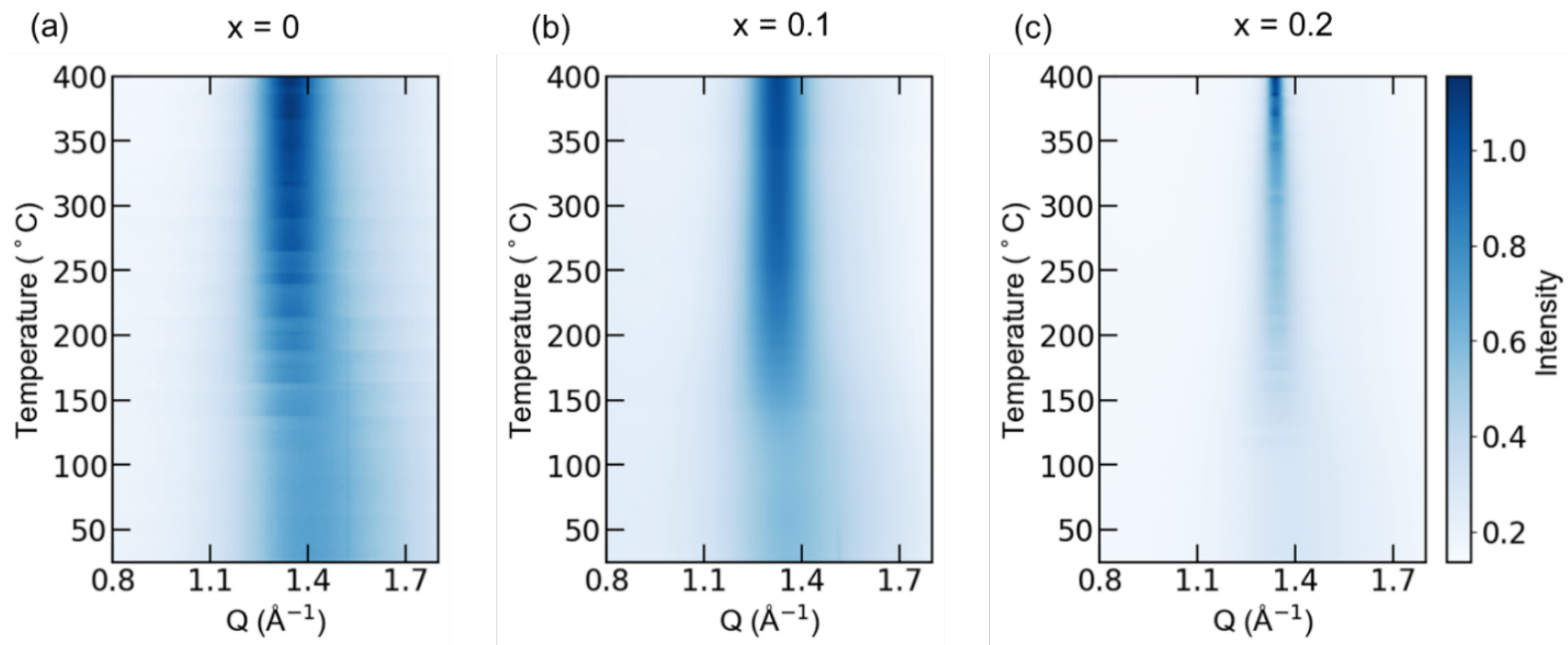

Figure S7. Contour plots of the *in-situ* synchrotron XRD data obtained upon heating $Li_{0.5}Mn_{0.7}Zr_{0.2-x}Ti_xO_2$ ((a) x = 0, (b) 0.1, and (c) 0.2) to 400 °C. Only the low-*Q* region is shown here. The phase transformation results in the appearance and sharpening of a new reflection at $Q \approx 1.35$ Å^{-1}.

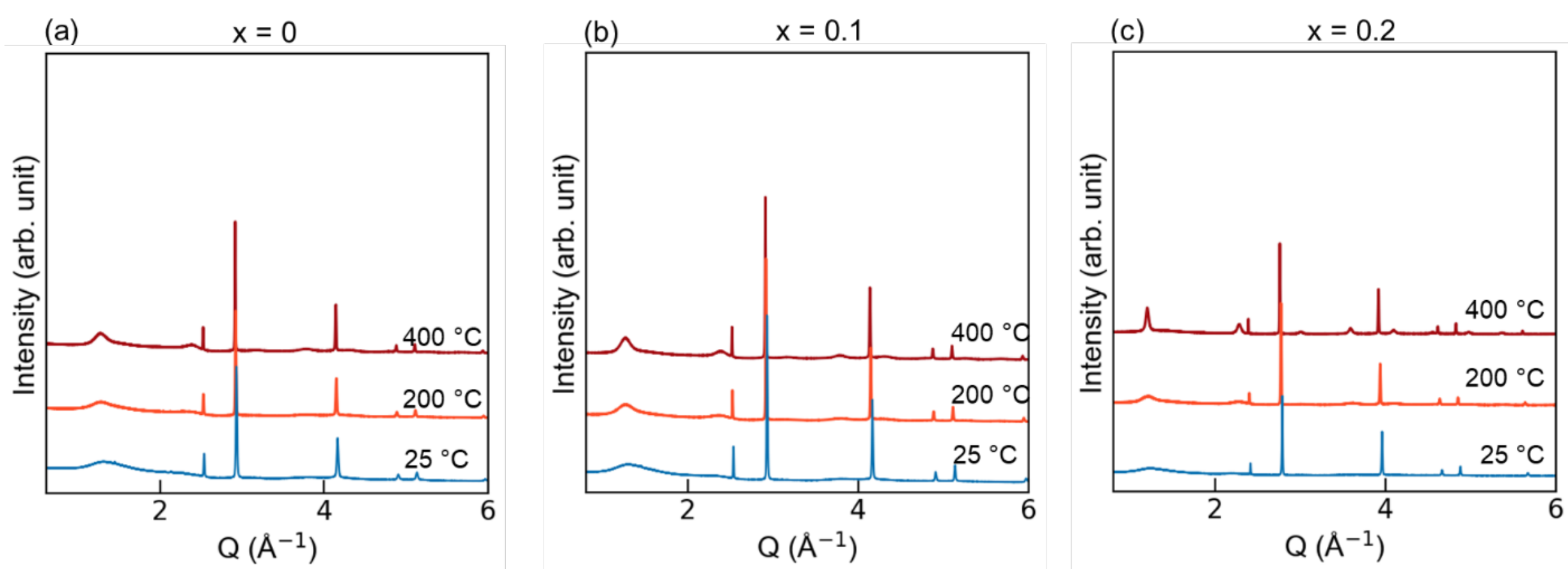

Figure S8. Synchrotron XRD data at select temperatures obtained upon heating $Li_{0.5}Mn_{0.7}Zr_{0.2-x}Ti_xO_2$ ((a) x = 0, (b) 0.1, and (c) 0.2).

Note S3 — In-situ Neutron PDF analysis on $Li_{0.5}Mn_{0.7}Zr_{0.2-x}Ti_xO_2$ equilibrated at different temperatures.

Neutron PDF data was collected on heating the x = 0, 0.1, and 0.2 compounds from 25 °C to 100 °C, 200 °C, 300 °C, and 400 °C, shown in **Figure S9-S11**. Samples were heated from room temperature to 400 °C at a rate of 10 °C/min. The temperature was held at 25, 100, 200, 300, and 400 °C for 2.5 hours at each step, comprising a 0.5 hour equilibration period followed by 2 hours of continuous data collection. For all temperature holds, individual scans obtained during the 2-hour data collection period showed negligible evolution, indicating that the samples had reached equilibrium after the initial 0.5 hour period. Therefore, the data was averaged over the full 2-hour data collection period to improve data quality. The magnitude of the structural changes taking place over the 25-100 °C, 100-200 °C, 200-300 °C, and 30-400 °C temperature intervals was quantified by taking the difference of the NPDF data collected at both ends of each temperature interval, normalizing, and summing point by point; the summed differences are presented in **Figure S12.** As expected, the most substantial structural changes are observed for $Li_{0.5}Mn_{0.7}Ti_{0.2}O_2$ and the smallest changes are observed for $Li_{0.5}Mn_{0.7}Zr_{0.2}O_2$. For the x = 0 and 0.1 compounds, the structural changes occur primarily below 200 °C. The limited apparent structural evolution beyond 200 °C could be due to rearrangements over length scales that exceed the resolution limit of NPDF analysis (~50 Å). In contrast, for $Li_{0.5}Mn_{0.7}Zr_{0.2}O_2$, the local structure appears to evolve over all temperature intervals, indicating that the structural rearrangements are short-range (within ~50 Å) up to at least 400 °C. These observations further confirm that Zr incorporation impedes the formation of larger spinel-ordered domains during the heat treatment.

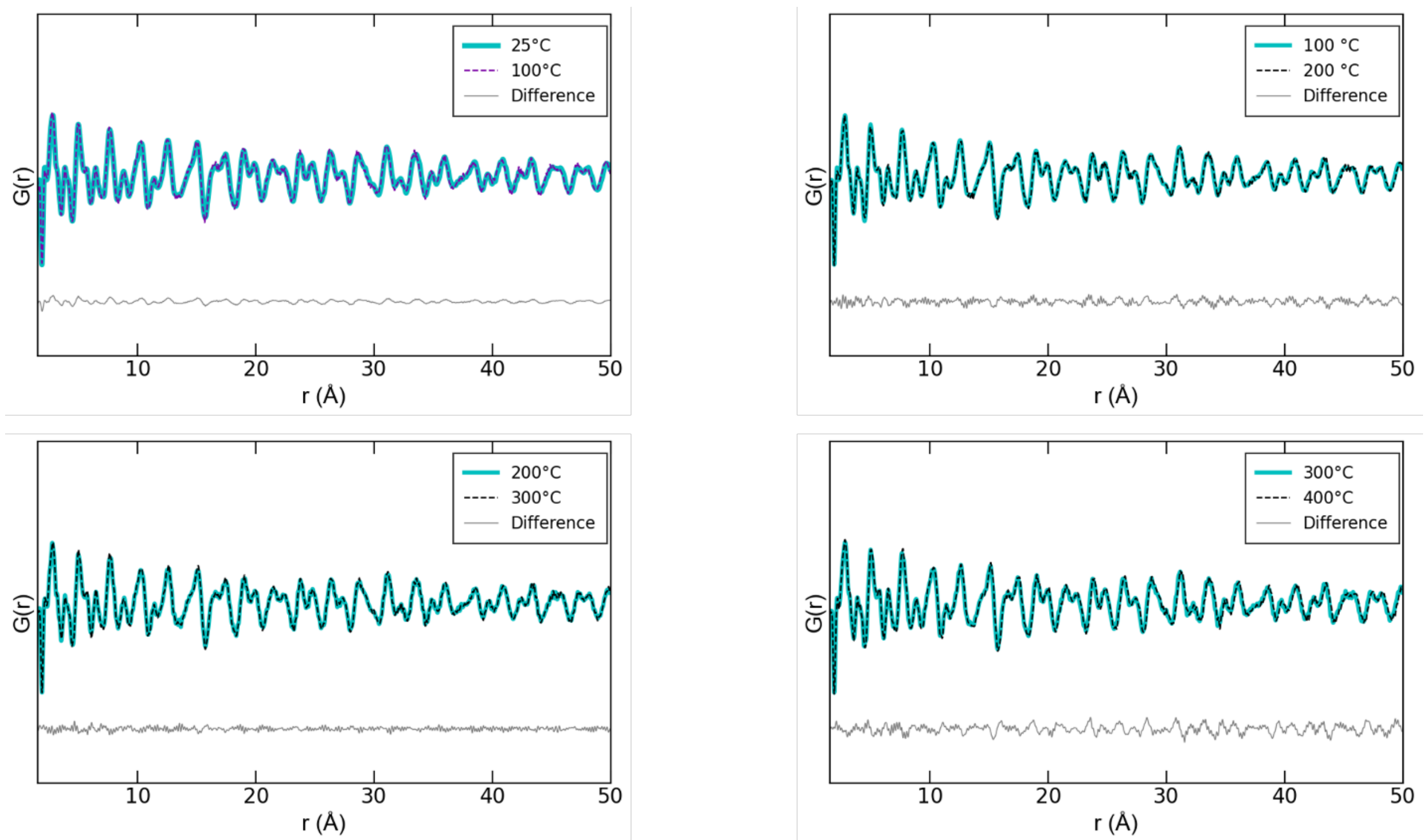

Figure S9. Comparison and difference of neutron PDFs of $Li_{0.5}Mn_{0.7}Zr_{0.2}O_2$ between adjacent data collection temperatures.

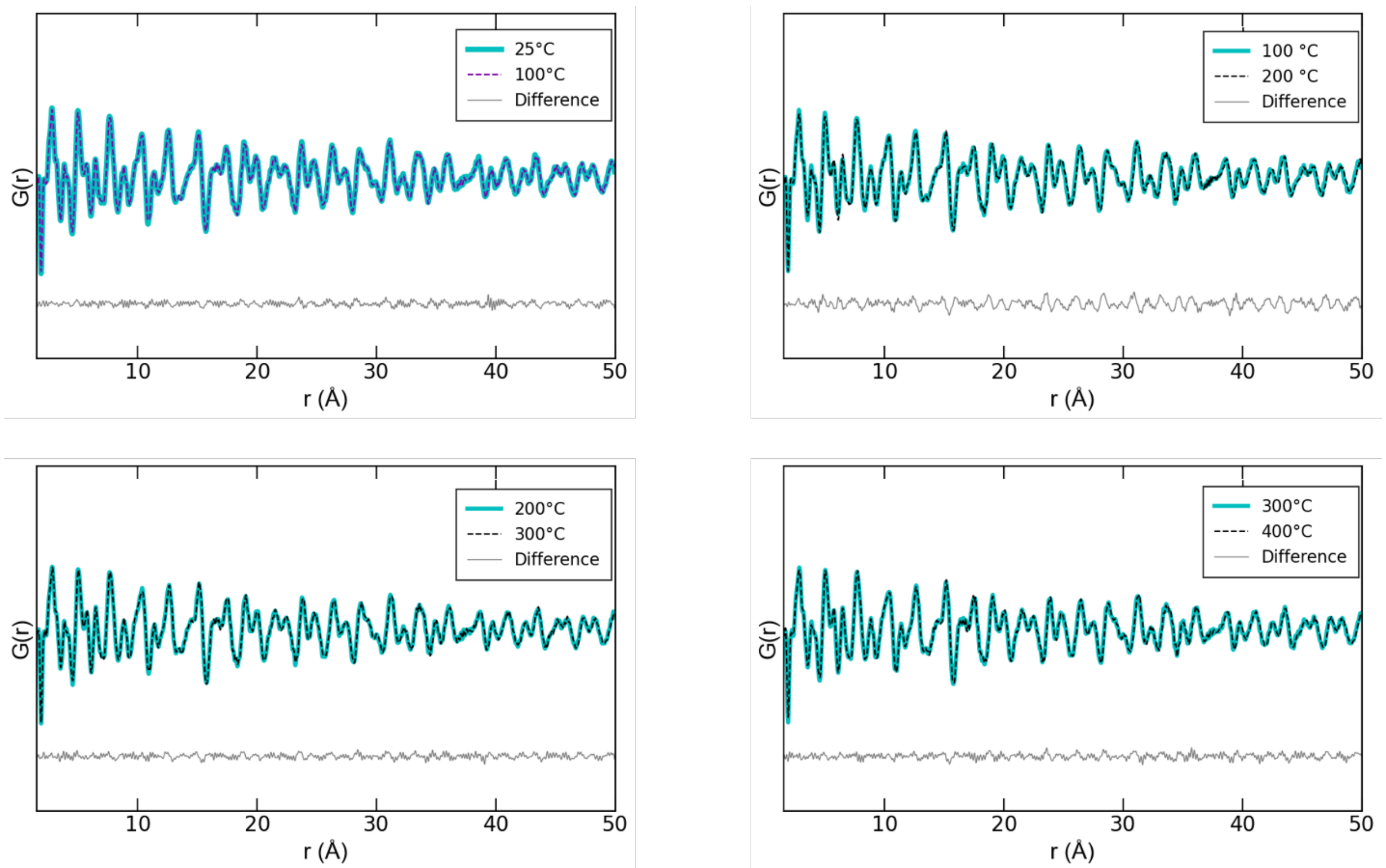

Figure S10. Comparison and difference of neutron PDFs of $Li_{0.5}Mn_{0.7}Zr_{0.1}Ti_{0.1}O_2$ between adjacent data collection temperatures.

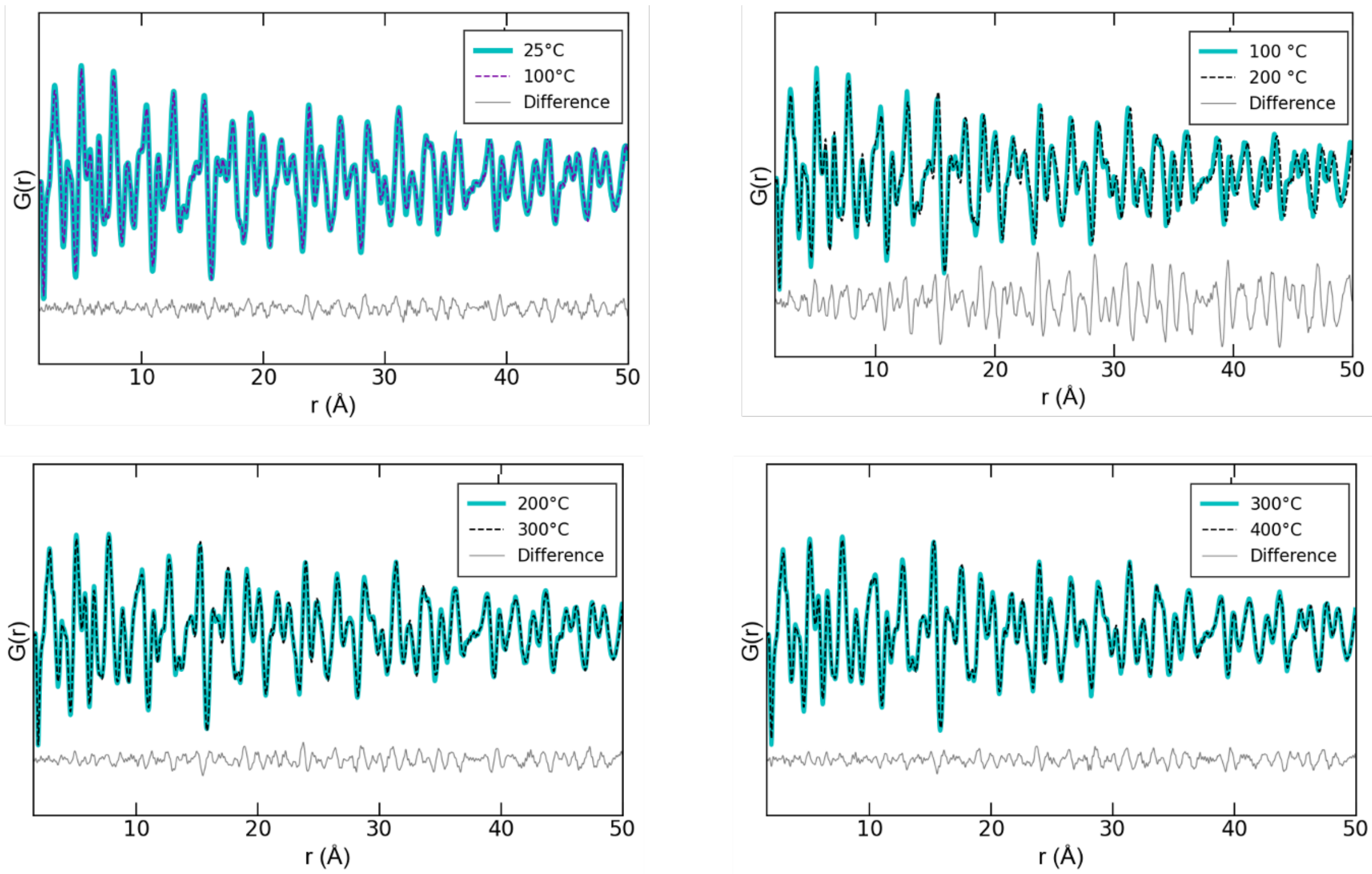

Figure S11. Comparison and difference of neutron PDFs of $Li_{0.5}Mn_{0.7}Ti_{0.2}O_2$ between adjacent data collection temperatures.

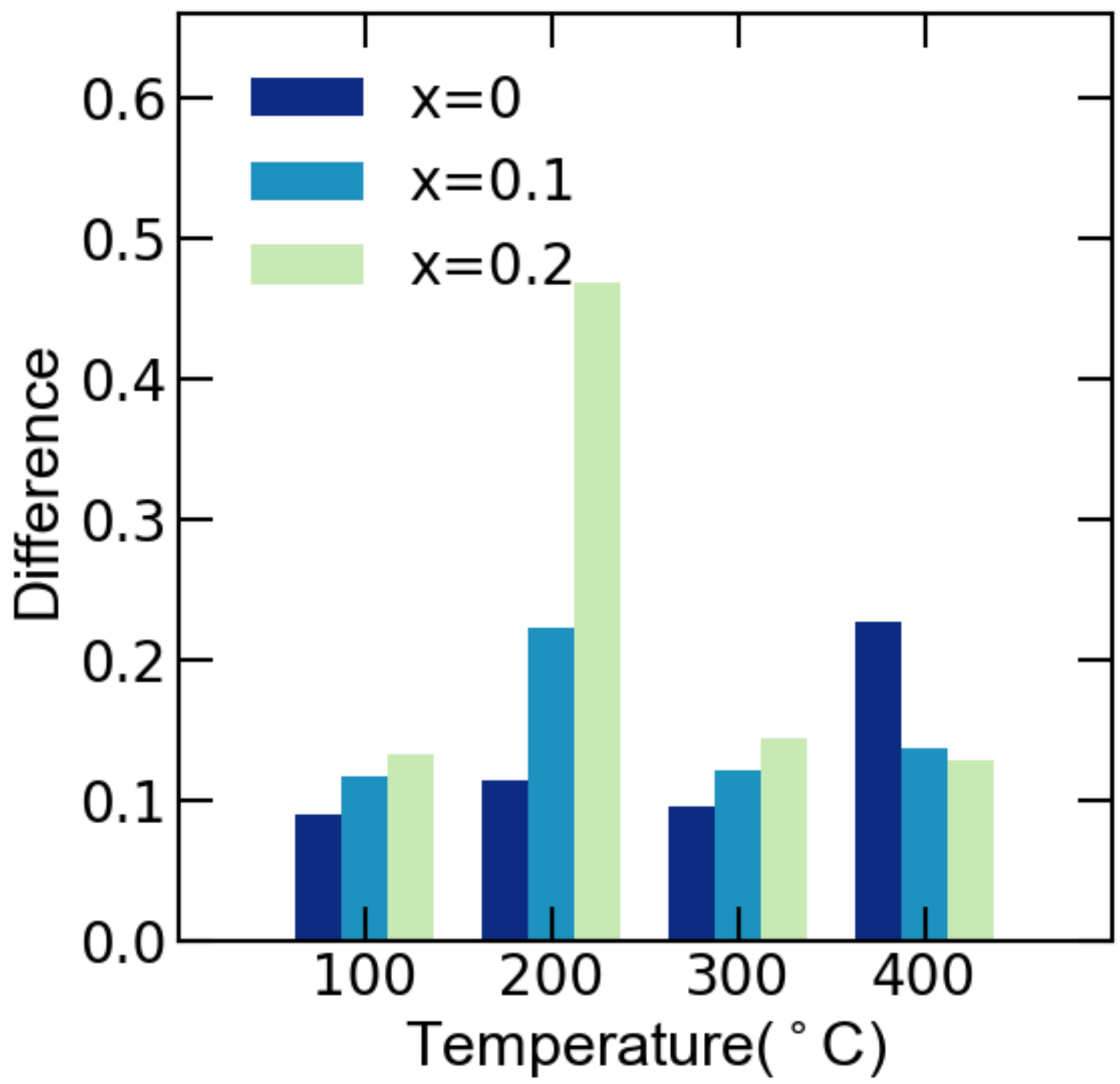

Figure S12. Summed difference of *ex situ* neutron NPDF patterns (**Figure S9**-**S11**) obtained on $Li_{0.5}Mn_{0.7}Zr_{0.2\text{-}x}Ti_xO_2$ samples at both ends of the following temperature intervals: 25–100°C, 100–200°C, 200–300°C, and 300–400°C.

Note S4 — Additional details on ^{7}Li solid-state NMR analysis of $Li_{0.5}Mn_{0.7}Zr_{0.2-x}Ti_xO_2$ (x = 0, 0.05, 0.1, 0.15, and 0.2) before and thermal activation

To better understand the impact of Zr and Ti on the evolution of the Li environments in $Li_{0.5}Mn_{0.7}Zr_{0.2-x}Ti_xO_2$, ^{7}Li solid-state NMR measurements were performed on the entire compositional series (x = 0, 0.05, 0.1, 0.15, and 0.2) before and after a heat treatment at 400 °C for 4 h. Before the heat treatment, the ^{7}Li NMR spectra obtained on the chemically-delithiated DRX samples comprise a broad resonance centered at approximately 700 ppm, as shown in **Figure S13**. A very weak signal is also observed at 0 ppm, which is characteristic of a Li-containing diamagnetic impurity and is tentatively attributed to residual Li_2CO_3 precursor or LiOH/Li_2O formed during the synthesis. We note that the diamagnetic impurity signals are underestimated in our spectra as our data acquisition parameters were optimized for the paramagnetic (cathode) signals and a 1 s inter-scan delay was used. The broad resonance results from overlapping signals due to a broad distribution of Li local environments in the paramagnetic (Mn-containing) cathode, as has been described in our previous work[8]. Its large positive shift arises from strong through-bond (Fermi contact) interactions between the ^{7}Li nuclear spin and unpaired electron spins from the *d* orbitals on nearby Mn ions. Specifically, the Fermi contact interaction depends on the delocalization of unpaired electron spin density from the Mn *d* orbitals, to bridging O *p* orbitals, and eventually to the Li *s* orbitals via so-called Mn-O-Li spin density transfer pathways. Hence, the magnitude of the overall ^{7}Li Fermi contact shift depends on the number and geometry (bond angles and bond lengths) of the Mn-O-Li interaction pathways. The 700 ppm shift observed for the chemically-delithiated $Li_{0.5}Mn_{0.7}Zr_{0.2-x}Ti_xO_2$ cathodes is in line with the average shift for octahedral Li in partially-delithiated

Mn^{3+}/Mn^{4+}-containing DRX cathodes, which is reported in our previous studies[9,10]. After the heat treatment, this broad resonance sharpens and, particularly for x > 0.1 systems, multiple sharper resonances can be distinguished. The reduction in linewidth arises from a narrower distribution of Li in the DRX cathode, indicating that Li becomes more ordered within the crystal structure. For $Li_{0.5}Mn_{0.7}Zr_{0.2-x}Ti_xO_2$ with x = 0.15 and 0.2, the magnitude of the average paramagnetic ^{7}Li chemical shift is reduced after the heat treatment, and a prominent resonance emerges at ~520 ppm. This shift matches that of tetrahedral Li in the $LiMn_2O_4$ spinel[11,12], suggesting that the structural rearrangements result in partial Li migration from octahedral to tetrahedral sites in the crystal structure. The increase in the intensity of the 520 ppm resonance with decreasing Zr content suggests that Zr impedes the local structural changes enabling Li migration to tetrahedral sites.

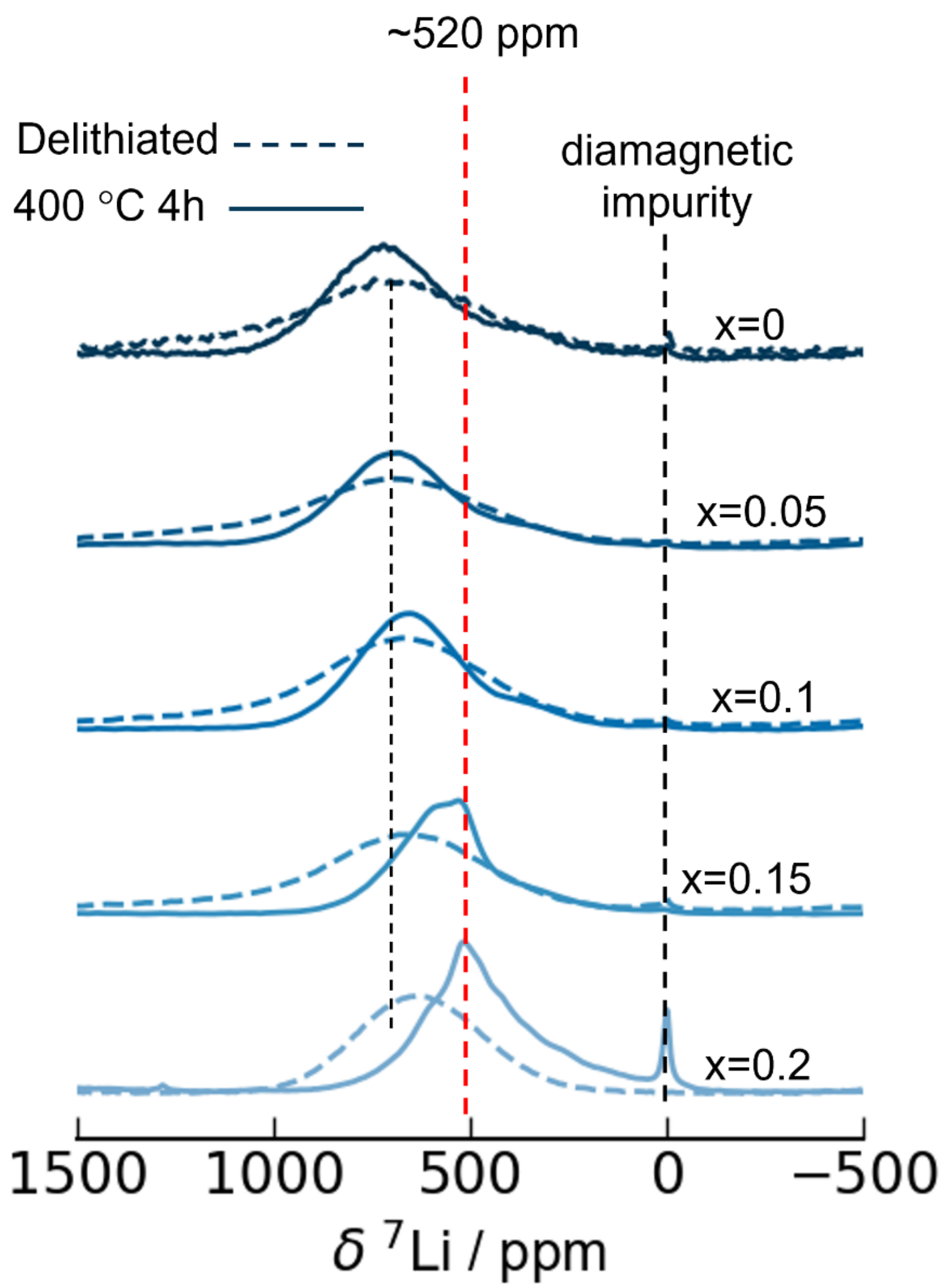

Figure S13. ^{7}Li solid-state NMR spectra collected on $Li_{0.5}Mn_{0.7}Zr_{0.2-x}Ti_xO_2$ samples before and after a 400 °C heat treatment for 4 h. Spectra are normalized by sample mass in the rotor and number of scans. Spectra were collected at 100 MHz (2.35 T) and under 60 kHz magic angle spinning (MAS), with a recycle delay of 1s. The signal corresponding to (a) diamagnetic Li-containing impurity(ies) is indicated with a black dotted line, and the central frequency of the Li environments emerging in the ordered, spinel-like domains is indicated with an orange dotted line.

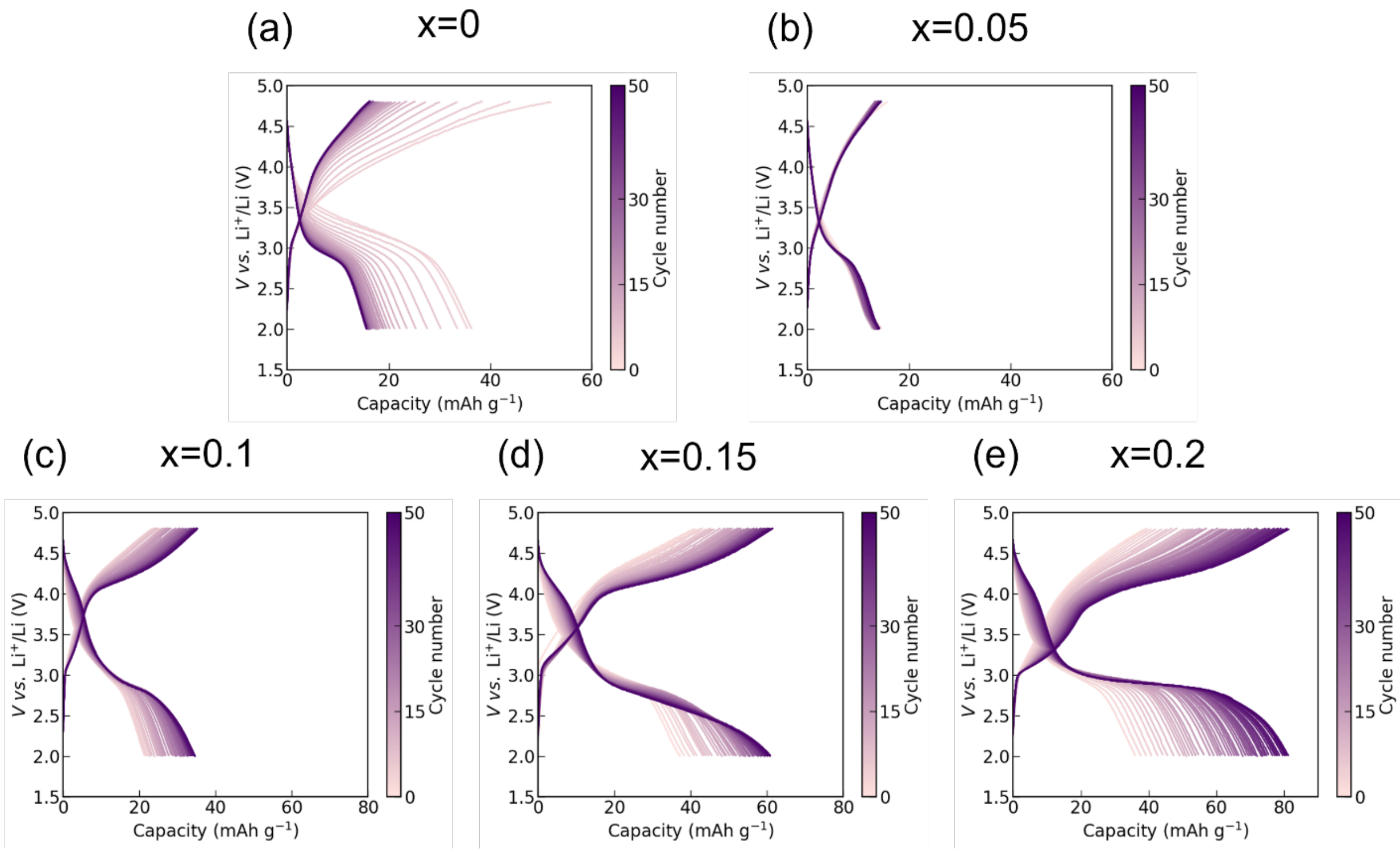

Figure S14. Evolution of the electrochemical profiles of as-synthesized $Li_{1.1}Mn_{0.7}Zr_{0.2-x}Ti_xO_2$ (a) x=0, (b) x=0.05, (c) x=0.1, (d) x=0.15 and (e) x=0.2 over the first 50 cycles, when cycled between 4.8 and 2 V vs. Li^+/Li at 20 mA g^{-1}. The first-cycle charge curve is excluded. Electrode fabrication consisted of hand griding the as-synthesized DRX powder with a carbon conductive additive and binder in 7:2:1 mass ratio.

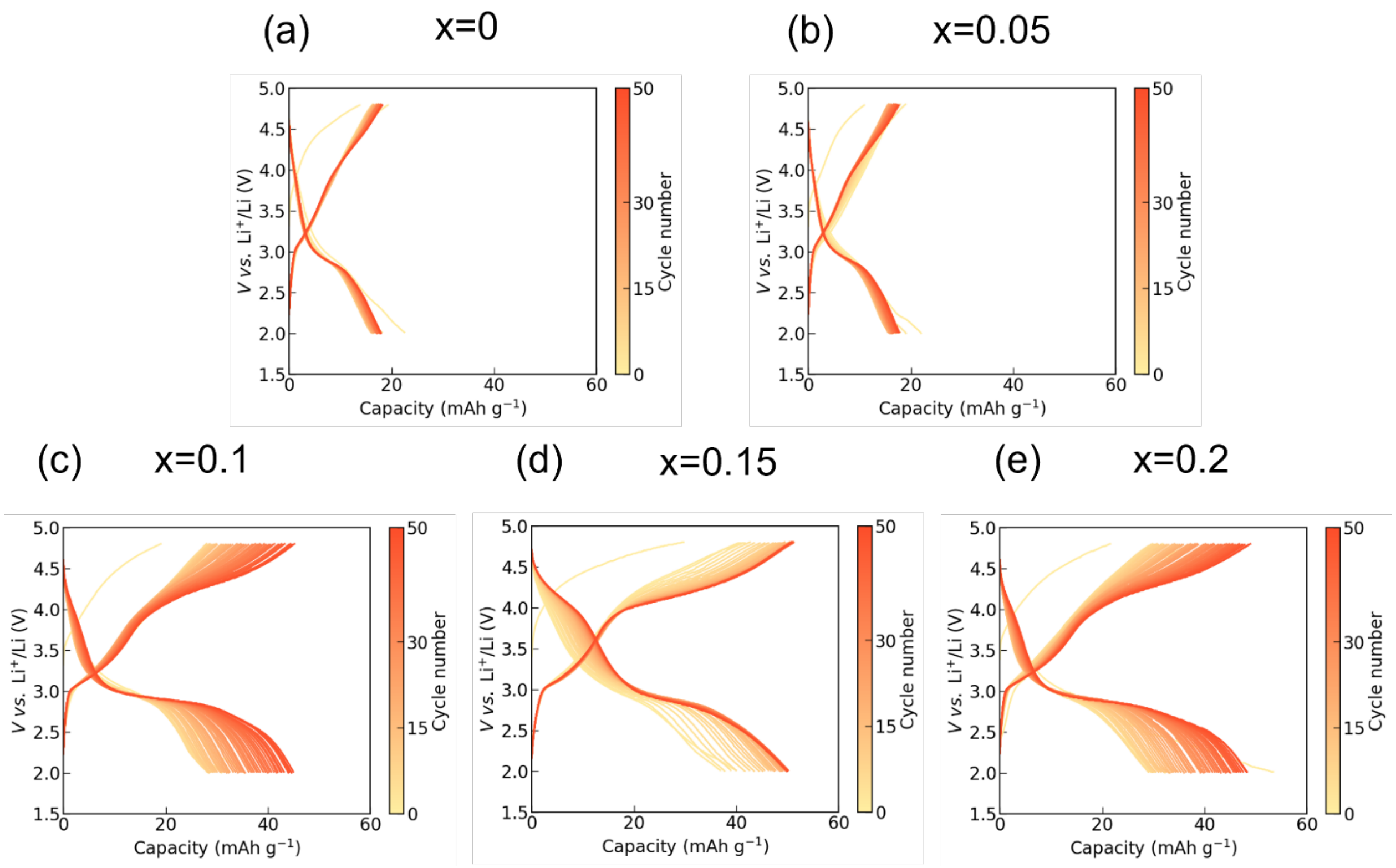

Figure S15. Evolution of the electrochemical profiles of chemically-delithiated $Li_{0.5}Mn_{0.7}Zr_{0.2-x}Ti_xO_2$ (a) x=0, (b) x=0.05, (c) x=0.1, (d) x=0.15 and (e) x=0.2 over the first 50 cycles, when cycled between 4.8 and 2 V vs. Li^+/Li at 20 mA g^{-1}. Electrode fabrication consisted of hand griding the as-synthesized DRX powder with a carbon conductive additive and binder in 7:2:1 mass ratio.

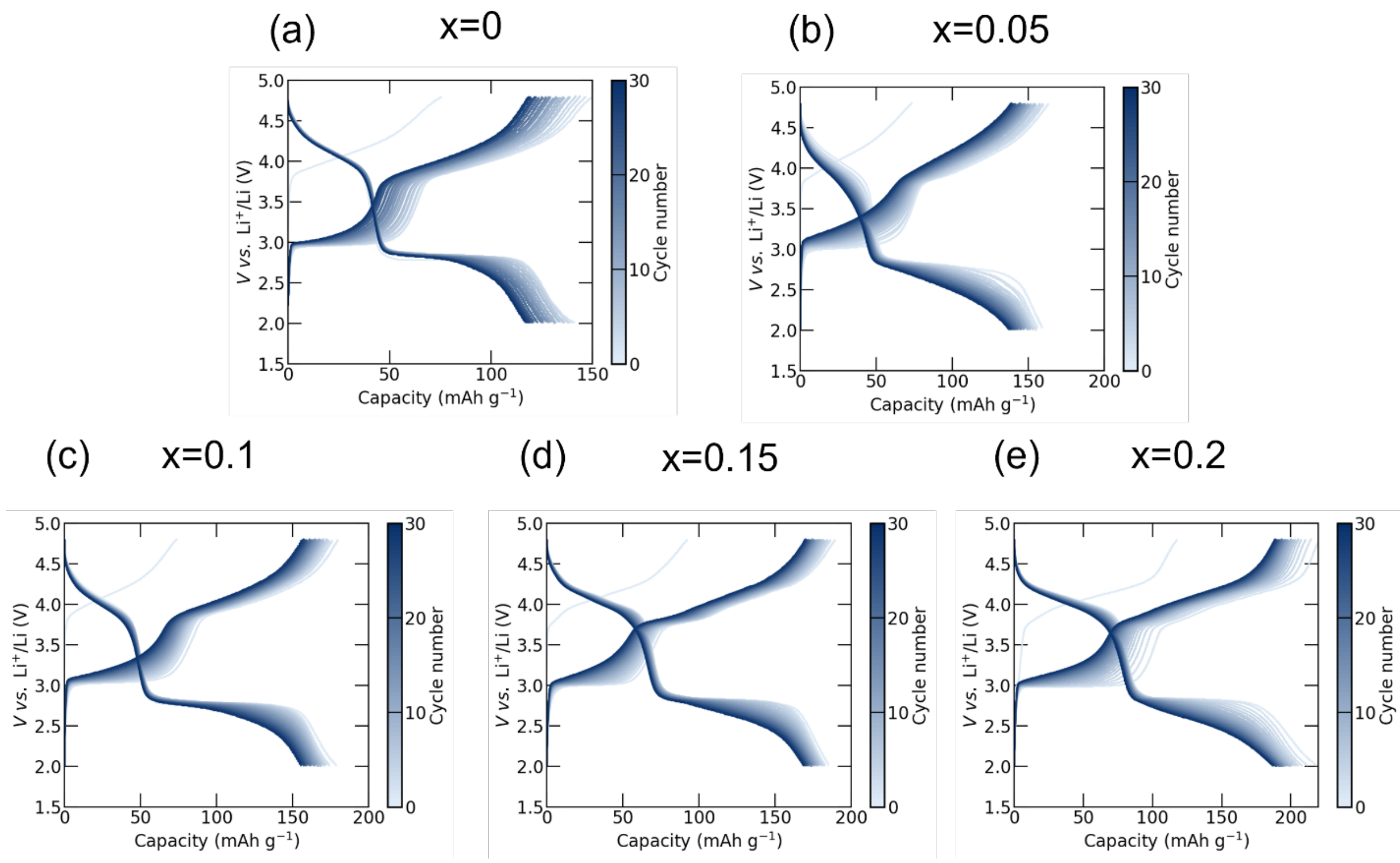

Figure S16. Evolution of the electrochemical profiles of chemically-delithiated and heat treated (400°C, 4h) $Li_{0.5}Mn_{0.7}Zr_{0.2-x}Ti_xO_2$ (a) x=0, (b) x=0.05, (c) x=0.1, (d) x=0.15 and (e) x=0.2 over the first 50 cycles, when cycled between 4.8 and 2 V vs. Li^+/Li at 20 mA g^{-1}.. Electrode fabrication consisted of hand griding the as-synthesized DRX powder with a carbon conductive additive and binder in 7:2:1 mass ratio.

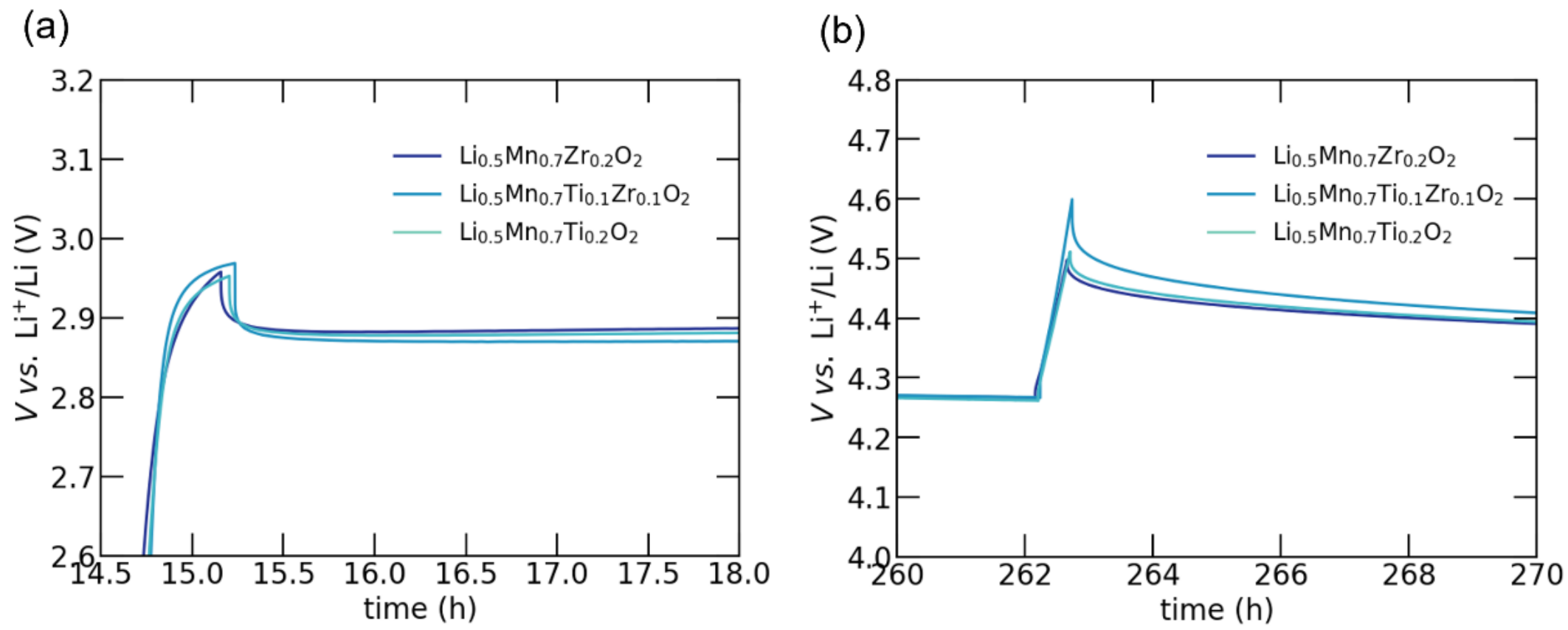

Figure S17. IR drop of thermally activated $Li_{0.5}Mn_{0.7}Zr_{0.2-x}Ti_xO_2$ (x = 0, 0.1 and 0.2) at (a) low voltage(~3V) and (b)high voltage(~4.5V) during the GITT measurement.

References

(1) Coelho, A. A. TOPAS and TOPAS-Academic : An Optimization Program Integrating Computer Algebra and Crystallographic Objects Written in C++. *J. Appl. Crystallogr.* **2018**, *51*, 210–218.

(2) Massiot, D.; Fayon, F.; Capron, M.; King, I.; Le Calvé, S.; Alonso, B.; Durand, J.; Bujoli, B.; Gan, Z.; Hoatson, G. Modelling One- and Two-dimensional Solid-state NMR Spectra. *Magn. Reson. Chem.* **2002**, *40*, 70–76.

(3) Ditmars, D. A.; Ishihara, S.; Chang, S. S.; Bernstein, G.; West, E. D. Enthalpy and Heat-Capacity Standard Reference Material: Synthetic Sapphire (Alpha-Al2O3) From 10 to 2250 K. *J. Res. Natl. Bur. Stand. (1934).* **1982**, *87*, 159.

(4) Navrotsky, A.; Kleppa, O. J. A Calorimetric Study of Molten Na2MoO4-MoO3 Mixtures at 970űK. *Inorg. Chem.* **1967**, *6*, 2119–2121.

(5) Navrotsky, A. Progress and New Directions in High Temperature Calorimetry. *Phys. Chem. Miner.* **1977**, *2*, 89–104.

(6) Navrotsky, A. Progress and New Directions in Calorimetry: A 2014 Perspective. *J. Am. Ceram. Soc.* **2014**, *97*, 3349–3359.

(7) Scharrer, M.; Bonatti, L.; Geraci, T.; Ushakov, S. V.; Majzlan, J.; Bustamante, M.; Kojitani, H.; Guo, X.; Xu, H.; Zhang, L.; Lilova, K.; Hayun, S.; Subramani, T.; Navrotsky, A. The Joys and Jitters of High-Temperature Calorimetry. *J. Am. Ceram. Soc.* **2025**, *108*, e20381.

(8) Giovine, R.; Yoshida, E.; Wu, V. C.; Ji, Y.; Crafton, M. J.; McCloskey, B. D.; Clément, R. J. An Experimental Approach to Assess Fluorine Incorporation into Disordered Rock Salt Oxide Cathodes. *Chem. Mater.* **2024**, *36*, 3643–3654.

(9) Cai, Z.; Ouyang, B.; Hau, H. M.; Chen, T.; Giovine, R.; Koirala, K. P.; Li, L.; Ji, H.; Ha, Y.; Sun, Y.; Huang, J.; Chen, Y.; Wu, V.; Yang, W.; Wang, C.; Clément, R. J.; Lun, Z.; Ceder, G. In Situ Formed Partially Disordered Phases as Earth-Abundant Mn-Rich Cathode Materials. *Nat. Energy* **2024**, *9*, 27–36.

(10) Li, T.; Geraci, T. S.; Koirala, K. P.; Zohar, A.; Bassey, E. N.; Chater, P. A.; Wang, C.; Navrotsky, A.; Clément, R. J. Structural Evolution in Disordered Rock Salt Cathodes. *J. Am. Chem. Soc.* **2024**, *146*, 24296–24309.

(11) Lee, Y. J.; Wang, F.; Grey, C. P. 6Li and 7Li MAS NMR Studies of Lithium Manganate Cathode Materials. *J. Am. Chem. Soc.* **1998**, *120*, 12601–12613.

(12) Pigliapochi, R.; Seymour, I. D.; Merlet, C.; Pell, A. J.; Murphy, D. T.; Schmid, S.; Grey, C. P. Structural Characterization of the Li-Ion Battery Cathode Materials LiTixMn2-XO4 (0.2 ≤ x ≤ 1.5): A Combined Experimental 7Li NMR and First-Principles Study. *Chem. Mater.* **2018**, *30*, 817–829.
